# Fostering Resilient Health Systems in India: Providing Care for PLHIV Under the Shadow of COVID-19

**DOI:** 10.3389/fpubh.2022.836044

**Published:** 2022-05-31

**Authors:** Neha Parikh, Angela Chaudhuri, Syama B. Syam, Pratishtha Singh

**Affiliations:** Swasti, Bangalore, India

**Keywords:** HIV, COVID-19, healthcare providers, resilience, health system

## Abstract

**Introduction:**

The novel coronavirus or COVID-19 has resulted in major human casualties, and extreme socio-economic crises causing catastrophic disturbances to health systems and communities alike. This study qualitatively explores the challenges experienced by healthcare providers while providing services to people living with HIV (PLHIV) during the pandemic outbreak and subsequent lockdown in India. The paper also explores strategies developed and adopted to provide continued care for PLHIV.

**Methods:**

Using an empirical phenomenological approach, qualitative in-depth telephonic interviews were conducted with 19 HIV care providers from five states in India. The recorded interviews were transcribed and analyzed using inductive thematic analysis with the help of Dedoose software.

**Results:**

From the analysis of participants' narratives, three main themes emerged: (1) Challenges of working during a pandemic; (2) Remodeling care delivery to ensure continuity of services; (3) Resilience.

**Discussion:**

Our findings highlight the challenges that providers faced, despite which, adaptive efforts were made to continue providing quality care for PLHIV through ingenious and innovative strategies. To foster resilient health systems, health workers are the primary stakeholders. We recommend formal social protection, comprehensive primary healthcare support, and sufficient capacity building for health workers for their self-care and pandemic preparedness.

## Introduction

The novel coronavirus or COVID-19, has resulted in major human casualties, and socio-economic crises causing catastrophic disturbances to health systems and communities alike. What followed was a shift in the priorities of governments and scientific communities to focus entirely on COVID, pushing everything else to the sidelines (1). To handle the immense strain caused by COVID-19 on health systems, equipment and providers were reallocated to the pandemic response. These changes adopted by nations posed a serious threat to the advances made in the control of diseases like HIV among others, by affecting the delivery of services as well as access to treatment and care (2). Care providers were asked to forego the ongoing, critical needs of People Living with HIV (PLHIV) which led to significant disruption in service delivery (3).

While the world debates and makes amends to equip health systems to battle COVID-19, there is a matter that is equally alarming, yet neglected - the wellbeing of health care workers (HCWs) and the impact of this pandemic on their physical and mental health. Healthcare providers (HCPs) have the highest risk for contracting the virus, because of the nature of their job- caring for infected patients and being in contaminated environments. Recent studies suggest that there is an increase in the prevalence of mental health problems among HCPs, especially frontline workers (4). The disproportionate risk of getting infected, along with a higher workload and inadequate working conditions might have a bearing on their psycho-physiological status (5, 6). Similar findings have been reported in studies conducted among HCWs during the 2003 SARS outbreak too (5). A recent study that compared mental health outcomes among medical and non-medical trained staff revealed that the prevalence of anxiety was higher among non-medical staff (7). HIV care providers where the workforce comprises medical and non-medical professionals like counselors, technicians, etc. might be particularly vulnerable to mental health problems as they operate in an intersecting space of COVID and HIV.

The ability to adapt and prevail in the face of adversities, or to be resilient is important while dealing with crisis situations. Findings from a systematic review indicate that resilience has a protective effect on negative stressors and mental outcomes like anxiety, depression, etc (8). While HCPs are known for their emotional resilience and fortitude, the challenges posed by this pandemic were certainly novel as the name suggested, making it difficult for providers to cope. Working tirelessly to provide care for a disease that spreads through human-to-human contact, fear of not knowing enough about the virus, and lack of a specific treatment certainly magnifies the stress of the providers and will in turn affect the quality of care (9, 10). A recent study among healthcare workers in Indonesia identified that higher levels of anxiety were significantly associated with lower levels of resilience (10).

While many sources affect the wellbeing of the health workers as part of their health system experience, it is important to identify these stressors and concurrent coping mechanisms to prevent further burnout as well as strengthen support mechanisms. This study aims to do a qualitative exploration of the challenges experienced by HIV care providers while providing services during the pandemic outbreak and subsequent lockdown in India as well as any solutions adopted to overcome the crisis.

## Methods

### Study Design

Using an empirical phenomenological approach, a qualitative study through telephonic in-depth interviews was conducted with health care workers providing care for PLHIV in five states of India. This research approach is considerably influenced by “phenomenology” which deals with the study of phenomena, how things appear or occur. Phenomenological qualitative research aims to understand experiences and meanings within the context in which a particular experience takes place (11). Therefore in-depth interviews using a semi-structured guide were used as the primary method of investigation in our study. The provider interviews were conducted as part of a larger study exploring the disruptions experienced by PLHIV while accessing sexual reproductive health rights and services during the COVID-19 pandemic and subsequent lockdown in India which occurred from March to May 2020 (12).

### Participants and Settings

The study was conducted with HIV providers from five states in India-Karnataka (KR), Andhra Pradesh (AP), Telangana (TS), Tamil Nadu (TN), and Maharashtra (MH). These particular sites were chosen since according to the recent estimation of key indicators for prioritizing HIV/AIDS programs in India, these were among the 15 top states where HIV-specific unmet needs were greatest (13). Further, these states have an adult HIV prevalence higher than the national average and were among the top six states with the highest mortality from AIDS (14). In addition, since coronavirus cases were steadily increasing at these places (15), and the strong professional relationship Swasti^1^ has with around 60 Community-based Organizations (CBOs) for over two decades, is what made us choose these five states as our study states.

Taking into account the experience CBOs have in working with health systems, especially those providing care for PLHIV, they were used to recruit participants in the study. Participants were sampled using purposive sampling with a maximum variation technique to get diverse opinions of challenges experienced by different health care workers. While the participants were recruited from different streams to diversify the sample, the interviews continued till no further information was coming forth from the participant narratives or till saturation was achieved. Only those individuals who have been providing services to PLHIV for at least 2 years were included in the study, to ensure that they had ample experience with the health system.

### Data Collection

Data was collected *via* in-depth telephonic interviews which ranged from 45 to 60 min. A semi-structured interview guide was developed and piloted with a few HIV care providers to test the validity of topics. Based on the observations from the pilot, the guide was modified and a final version of the tool was made, after deliberate consensus within the research team. This tool was then translated into the local language for ease of data collection.

The interviews were conducted by a four member research team from August to October 2020, just after the first wave was considered to be over. Two researchers were there on each call (one as interviewer and one as moderator and to take notes), along with the participant. Before commencing the interview the purpose of the study was explained to the participant in detail and verbal consent was obtained for their voluntary participation as well as recording the interview. Even though the lockdown was over in some of the states during the time of conducting the study, there were still restrictions in many parts of the states which affected the movement and access to health care for communities, as well as providers. Keeping this in mind, we encouraged the participants to share their experiences of disruptions during the official lockdown and the period after. Interviews were conducted till data saturation was obtained, a point where no further information emerged from the participants' narrative of their experience.

### Ethical Considerations

Since the study involved human participants, all procedures were carried out per the ethical standards of the Catalyst Group Institutional Review Board, and ethical approval was taken from the same. Study participants were informed telephonically about the study details, as well as any possible risks before data collection. Only once informed consent was obtained, the data collection procedure began. Providers were also told that they could stop the interviews at any point, and skip any question which they weren't comfortable with. Furthermore, high levels of confidentiality were ensured with strict measures taken to protect the respondents' identity. Although participant names and the details of the facilities they work in were asked for the interviews, once transcription was done, codes were given to each participant so that their identity remained masked. Any identifiable information of theirs would not be disclosed in any report, research paper, or presentation. Moreover, although consent was taken to record the telephonic conversations, these recordings would not be shared with anybody other than the research team and they are stored in password-protected drives with limited access.

### Data Analysis

All the interviews were transcribed into English by a team of transcriptionists and 25% of the transcripts were randomly cross-checked with the original recordings to ensure the quality of the transcriptions. The transcripts were uploaded to Dedoose - a software used to support qualitative data analysis and was analyzed using “inductive thematic analysis.” The research team underwent in-depth training on how to use Dedoose to code, analyze and export data from the transcripts.

For analyzing the data using thematic analysis, the team used the guidelines put forth by Braun and Clarke (16). The first step in the process of analysis was familiarizing with the data, which involved reading the transcripts multiple times and making extensive notes on the researcher's initial thoughts and what patterns have been identified in the data. In the next step, codes were generated based on certain features of the data like commonly occurring patterns in the transcripts or main points that were discussed in each interview. Using Dedoose, phrases, sentences, and paragraphs were highlighted and given tags to match the codes. Once the entire dataset was coded like this, the team met virtually to discuss and review each other's coding and continued to the next phase of generating themes. From the codebook generated from Dedoose, the codes were grouped together based on similarities and differences. At this point, some codes that were not particularly relevant to the phenomenon that was being discussed were discarded. Once the teams were in agreement on the final list of themes and sub-themes, the themes were given clear names and succinctly defined to convey what they represented and how each of them fit into the larger narrative. The final stage of the analysis was detailing everything that happened as part of the study, right from the introduction to the final narrative of results, discussion, and conclusions which are discussed in different sections in this paper.

## Results

A total of 19 participants from five states, shared their experiences of providing care during the COVID-19 pandemic and subsequent lockdown, as well as the mitigating measures adopted to ensure service delivery. Among the participants, we had four nurses, four doctors, four program personnel, five counselors, and two field workers. On average, the providers have been working with the PLHIV for 11 years. The mean age of the participants was 42 and the sample included nine females, eight males, and one transwoman. Ten were working in the public sector and nine were from the private sector.

From the analysis of participants' narratives, three main themes emerged: (1) Challenges of working during a pandemic; (2) Remodeling care delivery to ensure continuity of services; (3) Resilience.

### Challenges of Working During a Pandemic

This theme details the challenges experienced by the providers at multiple levels while providing care for PLHIV during the pandemic and subsequent lockdown. The challenges are described in three separate sub-themes—systemic, community level, and personal.

#### Systemic Disruptions in Service Delivery

The first subtheme describes the disruptions that occurred in the HIV healthcare sector and how that affected the service delivery as well as access to care.

Providers reported that the facilities were suspended for months together, partially and sometimes completely due to the stringent lockdowns in all of the states included in our study. There was a considerable delay, and sometimes an almost complete cessation in the provision of routine healthcare services including supportive care, as well as routine screening and testing. Even when supplies were available, the unavailability of transportation and the unwillingness of staff to travel, due to fear of COVID-19 resulted in a delay in procuring the necessary supplies.

“*Earlier, on an average 100–150 clients will meet one counselor each day for proper counseling. Due to lockdown, there was no treatment provided, and counseling did not happen too.” - 50, ART Counselor, TN*

“*For the first few months, we didn't have proper supply. Funding was there. But to bring the available resources, we didn't have proper transport, human resources...staff was not willing to go to far off places.” - 47, ART Doctor, KR*

The priorities given to the healthcare staff for handling the pandemic situation interrupted the service delivery in all aspects—funding and equipment got diverted, providers got shifted to COVID duty, and hospitals became COVID-19 care centers. As a result, providers were not able to care for any illness other than COVID-19 and had to reject help to patients on multiple occasions.

“*Our entire lab was converted to COVID-19 virology so they closed it all down. The CD4 machines, both the lab technicians were officially assigned to Covid duties...So, lab technicians are very important to us... They did the duty for more than 6 months...so the testing completely stopped…It completely stopped.” - 35, ART Doctor, AP-TS*

“*There was no admission here at the health center for PLHIV patients facing difficulty at any time even during the lockdown due to COVID-19 care. PLHIV people still are not allowed admission in GH, they are facing trouble getting care as COVID-19 cases or treatment is the main priority.” - 41, Program Staff, TN*

The majority of the providers, with the exception of a few, mentioned that while there was a clear directive to ensure continuity of services for PLHIV, there was a general lack of planning and preparedness to handle the crisis situation, in terms of arranging transportation, allocating budget and other resources, information communication, etc.

“*We had a disruption in the transportation of medications… Though the papers and everything said the Postal Service has opened up and will take the essential drugs, it was for diabetes, heart disease, etc*.

*Nobody mentioned anything about PLHIV.” - 54, Primary care Physician, KR*

“*Sir, if the office had arranged vehicles for us to travel, it'd have been very easy for facilitating services. But it was not there.” 45, Outreach Worker, KR*

“*This issue of shortage of staff is omnipresent. We see this problem in all the departments. This is due to less manpower. In the initial days, we found it quite difficult. Firstly, we had to finish our work, and then we were asked to perform the call duty for COVID counseling. Then we were placed in the COVID centers, to counsel COVID and non-COVID patients.” - 42, ART Counselor, MH*

#### Patient and Community Level Setbacks

The second sub-theme describes the challenges faced by the providers because of the effect of the pandemic on the care-seeking behavior of PLHIV communities.

The unavailability and unaffordability of transport services along with the crippling fear and exacerbated stigma that surfaced with COVID-19, resulted in the majority of PLHIV refraining from seeking care. A considerable number of respondents also mentioned that the PLHIV were hesitant to seek care because of health centers becoming COVID care centers.

“*PLHIV could not turn up and have tests due to lack of proper transport, some could not have masks and condoms….Those living in rural areas could not come to the center and avail medicines.” 49, ICTC counselor, AP-TS*

“*Yes madam, they are well aware of the consequences of missing the medicine but they are ready to take that risk to protect their identity. They will say that they will somehow manage to find some money and come back in a month and buy medicines from here itself.” 39, ART Nurse, KR*

Another important challenge was the changes in the attitudes and behaviors of patients. Due to the restrictions that were implemented in the health centers, it was reported that the patients were not trusting the providers completely and stopped disclosing their health problems as they used to do before. Few providers also reported that the patients themselves limited their interaction with providers out of fear of getting COVID-19.

“*The usual process of sitting next to the doctor and analyzing the conditions of the patients is not happening... Now they just have to go there, collect the tablets and leave the place. They don't have any freedom to talk to the doctor as they had done before or they can't even tell their own experiences. How could they be standing behind the ropes and shout out their experiences? They might feel bad right? It is very different when we talk next to the person and speak when compared to sending it through a middleman. Now even if they had white discharge they feel discomfort to say it out so they don't tell it.” 34, Outreach Worker, TN*

“*The patients were scared and they wanted to leave as soon as possible by just wanting to take the medicines and go. They have the fear that looking at others and being with others, there is a possibility of catching COVID so the patients themselves were in a rush.” 44, ART Nurse, TN*

While providers resorted to community outreach or linking PLHIV with other centers, to ensure the availability of medicines, they faced a multitude of challenges that affected service delivery. Providers reported that fake credentials (name and address) provided by PLHIV made it difficult for the providers to identify the patients. There was also the constant worry of patient confidentiality getting breached by the presence of providers in the community. It was also reported by few respondents that the communities were not always in favor of outreach as they perceived the providers posed a far greater risk for them.

“*When I went looking for a person, their name was registered as a female name; it was not the original name. So I tried to say other things to find him. Once I found his house I went and tried calling him but his phone was switched off. Later I gave the medicines to his cousin and informed him to call back once he gets back home. He conveyed thanks for the effort we put to give him the medicines. But it was really challenging for me because people gathered around when I went to his home and then I realized that I shouldn't do this and put their identity at risk.” 50, ART Counselor, TN*

“*They also used to think that since we roam around a lot, what if they get infected from us? One group called me and asked, ‘How could you test other people, we should test you first' and asked me how could you come to our area like this?” - 42, NGO Staff, MH*

#### Personal Challenges

This sub-theme discusses how the impact of the pandemic on the provider as an individual affected the provision of services.

Like any other section of the society, providers also reported challenges in traveling during the pandemic making it difficult for them to reach facilities. Some providers, especially women and those with special needs mentioned that this became particularly difficult when they were providing outreach. If they did not have their own vehicle, they had to depend on other modes of transportation, and most of the time it was not available, forcing them to walk long distances or settling to pay higher charges. Another major concern shared was their experiences of interacting with police. The majority of the providers mentioned that the police were not very cooperative which led to delay in field operations as well as some degree of fear, especially in women providers.

“*...We faced a lot of issues during the lockdown. We had to explain to the police for 2–3 hours as to why we needed to go out and deliver these medicines and yet they were not convinced… It was very annoying*” *42, ART Counsellor, MH*

“*It was quite difficult for us to reach the center. I am an orthopedically handicapped person. It was tough for me to even get an auto at home. In addition to that, I had to pay much more for transport. But I still continued to serve.” 49, Counselor, AP-TS*

Because of the priority given to COVID care during the pandemic situation, there was a sudden change in the routine of providers. Providers reported that they didn't perform the usual routine tests and limited their interaction with the patients because of the limited staff availability as well as the preventive protocols. Providers mentioned that not having enough staff didn't just add to their workload but also affected the quality of care and in turn affected their satisfaction.

“*Right now we are on Covid duty. Initially, when we all were there it was easy to provide quality time for patients but now due to limited staff they can't be given more time and are sent back soon.”* 24, ART Counsellor, KR

“*I felt bad about not doing tests. CD4 testing is necessary for issuing and dosage of drugs. We couldn't do it due to less staff. They took our lab technicians for COVID needs. Job satisfaction was lacking. So I did feel bad about it as it was a direct order from our superiors.”* ART Counselor,

Having to handle dual responsibilities of caring for their usual patients and managing COVID duty and battling the environmental challenges took a serious toll on the provider community. The majority of them mentioned that they were burnt out and exhausted at the end of the day. Moreover, wearing heavy gear PPEs, which is not something they are used to, also caused a great deal of physical discomfort for them. It was also reported by a few providers that there was a shortage of PPE in some locations which meant they kept reusing the same items again and again.

“*It's the burnout which is happening because people are also tired of it. It's not that they don't care or they care less for PLHIV, but it's just that they cannot manage, they cannot do so much. They were overloaded with work, lots of patients coming with Covid, shortage of healthcare workers whether it is doctors, nurses, everybody was overburdened with work.” 54, Primary care Physician, KR*

“*It is very difficult with masks because we are constantly wearing them. From the time we come in the morning to the time we leave, we are continuously wearing masks. I feel that we do not get enough oxygen also.”* 46, Staff Nurse, AP-TS

“*We have to wear gloves to see them and examine them, we have to change them frequently but we had to manage with a pair of gloves for 3 days. We used to disinfect them and the next day also I used to wear the same gloves….Because for the first few months we didn't have a proper supply. Transport was not there. So, the hospital also had little supply.”* 47, ART Doctor, KR

Providers also mentioned that while they did all the good work, they didn't feel recognized or rewarded appropriately by the authorities. Many participants mentioned that on occasion they didn't even get their remuneration on time. The lack of any government support, discontinuance of previous provisions like travel allowance (TA), along with the additional expenditure to facilitate outreach made it extremely difficult for some of the providers to even afford their bare necessities.

“*4 months have gone and now 5th month is going on, still, no payment has been done yet due to Covid only.”* 42, NGO Staff, MH

“*Initially, even the front and second-line health workers were also provided TA but now it's completely stopped during this pandemic.”* 24, ART Counsellor, KR

“*... We are not even being recognized properly… Despite performing such crucial roles, governments don't recognize our service and give the salary we deserve.”* 49, ICTC Counsellor, AP-TS

“*Even in the PLHIV community, they're getting 2000–3000rs as a pension. Similarly, we also want such remuneration, madam. Please get us that. And we have very much difficulty in obtaining ration also since covid arrived.”* 45, Outreach Worker, KR

Many participants talked about their helplessness with the whole situation as they had no control over what was happening back then. Providers mentioned that they remember being in a quandary when they had to refer patients who became COVID positive to other facilities as they were not equipped to handle these cases. It was also discussed that they felt powerless over the outcomes and went into despair when they moved heaven and earth to ensure patients received care but still could not save them.

“*We had to refuse a few referrals who came in a bad situation because we were not COVID authorized hospital and we couldn't know whether that person is COVID positive or not I know of one woman who we sent to Gandhi hospital referral from the place where she came and we came to know that she died. The whole organization felt very bad about that.” - 50, Private Physician, AP-TS*

“*It's just very disheartening because you're losing your own patient, somebody you have been managing. How much ever we try and say you know we should not sympathize or empathize, but at some point it gives pain. Why did you go that extra mile? Just imagine, everything you do to make the patient survive and you still lose the patient, it's terrible.”* 54, Primary care Physician, KR

Finally and most importantly the providers mentioned that they were constantly worried about their susceptibility to COVID-19. While they performed their duty out of obligation and passion, they always had a fear of whether they were putting their loved ones or patients at risk of COVID-19.

“*They would call me inside but the thought that comes to my mind would be I have to maintain distance from them because we are exposed to so many places and people like GH, so because of us what if they get infected!..... Or if I get it from them then I am putting my family at risk. Both the ways it is problematic.”* 34, Outreach Worker, TN

“*And there is this thought about am I at risk, am I at risk, so it's anxiety. Until we reach our house, we cannot say that on the way who will have what, and whether we will possess the virus or what, we won't know. So, every time we have to be on the side of caution. In the house also we have to maintain social distancing with elders and children.”* 47, ART Doctor, KR

### Remodeling Care Delivery to Ensure Continuity of Services

The second theme describes the adaptations to the existing HIV care delivery model as well as the novel strategies adopted by the providers to overcome the challenges experienced during the lockdown and ensure continuity of services.

The majority of the providers reported prioritizing an uninterrupted supply of ART medicines over physical consultations or in-patient visits at health facilities. Taking into account the difficulties PLHIV experienced in reaching health facilities due to financial difficulties and lack of transport as well as their susceptibility to increased risk for COVID-19, community dispensing was widely adopted. To this end, providers conducted extensive outreach, collaborated with local health facilities, NGOs, and other community organizations to provide services for PLHIV. Providers reported informing the PLHIV in advance and dispensing the medicines and supplies at a community outreach point that was comfortable for the patients. Providers have also reported that they made every effort to ensure confidentiality of the patients' identity and condition.

“*Now patients are coming, but many of them could not come, then. Due to Covid, buses and all were stopped..., even if a patient came with their own vehicle, they would face problems. That is why we made a plan along with the NGOs and arranged for a truck... First we would do a follow-up on the phone and make a list of the medicine they needed, and a place would be decided. Then we would give them the information that we are giving drugs at this location, you can go there calmly and medicines will be given to you without any problems. Then, our truck would go there. It was like home delivery then sir.” 46, Staff Nurse, AP-TS*

The process of outreach was not just limited to dispensing of medicines, but also providing home visits to assess any illness as well as providing supportive care like counseling, etc. Wherever possible, providers also reported supplying communities with provisions and other supplies necessary for their basic sustenance too.

“*The meds were supplied through the ART counsellors only, so they counsel them and then after proper education the PLHIV patients will be given the meds.” 37, Program Manager, TN*

“*Most patients come to us with their problems as they have no work or no money during lockdown. We provided help through NGOs by giving them ration kits, coordinated with NGOs, ORWs, and ICTC care providers and provided medicines to patients' homes, who had difficulties to come to the ART centers.” 24, ART counsellor, KR*

Teleconsultation was another important service delivery adaptation reported by the majority of the respondents. This included reaching out to patients to provide a consultation for acute and chronic illness, psychosocial support as well as to facilitate appropriate care from nearby centers to which they were referred. In addition, providers also continued tracing of patients missing appointments or due for ART refill, etc. *via* phones instead of the usual physical follow-up.

“*So, anybody who had the symptoms of fever, cough, cold we consulted over the telephone. On a video consultation, you can really see whether the patient is actually dyspneic or not, whether they need to be taken to a hospital or managed at home. So, we did manage quite a few patients over the tele-consultation but then slowly by the 2nd week of April, we started resuming our homecare” 54, Primary care physician, KR*

“*We provided our ART centre contact number to all the patients in their green books, so most patients themselves contacted us during emergency, if not at the end if any patients were left out to buy medicines, we contacted them. All the patients were referred to taluk hospitals for their convenience, very few patients could afford to come here, we treated them.” 24, ART Counselor, KR*

Where PLHIV were able to reach ART centers or other care facilities, providers encouraged community members to seek services directly from health facilities. It was also reported that providers reached out to patients to inform them in advance about the availability and duration of services. In addition to following recommended Infection Prevention and Control practices in the centers, providers adjusted the patient flow and limited the duration of interaction to avoid overcrowding and thereby limit exposure to patients as well as healthcare staff.

“*Patients had a doubt whether the ART centers will be open or what, but we called them up and we have informed them that ART centers are not closed, you can come anytime before 5:00 o'clock and take the tablets.” 47, ART Doctor, KR*

“*The hospital has taken care to screen all patients before they enter and to maintain the social distance. Once they are admitted, the organization has seen that not many people were exposed.” 50, Private Practitioner, AP-TS*

“*Only critically ill patients were allowed inside the hospital to prevent exposure. Very few patients were treated during that time...Doctors treated patients in the same way they treated before COVID-19, they check patients and give them treatment but with precautionary measures” - 24, ART Counselor, KR*

### Resilience

The final theme details the different mechanisms identified and adopted by the providers to remain resilient in the face of the COVID-19 crisis.

#### Personal

This subtheme highlights the solutions the providers adopted, despite the multitude of service delivery and personal challenges they encountered while the coronavirus continued to spread.

Since they had no formal work policies which focused on their mental health, some providers took it upon themselves to reduce their stress levels and keep their emotional wellbeing in check. This was done by practicing meditation daily and holding frequent video calls between the team members to talk about work. Few providers also mentioned the support they received from their friends and family, how this developed their resilience and gave them the strength to keep working during this adversity.

“*Everyone was doing meditation during the lockdown so that in this stressful situation we have 10 mins of meditation for mental peace. What used to happen was that you get irritated when another person or staff isn't able to understand me, so that meditation was really helpful in controlling tough situations.”* 42, NGO staff, MH

“*I should also say that, I had strong support from my family - my husband and my kids. They knew the importance of my profession and supported me always.'*' 46, Staff Nurse, KR

“*Luckily our [supervisor] used to call us, and sessions were there to guide us and videos were exchanged between us so our stress was lowered.”* 42, NGO Staff, MH

Furthermore, since providers had a healthy and friendly relationship with their patients from the HIV community, having work satisfaction proved important for their psychological wellbeing. Some respondents from our study mentioned that getting the opportunity to work during the pandemic and help their communities made them happy and built their resilience. Providers also maintained constant communication with different health facilities and personnel working in the same field, so that they could coordinate and streamline service delivery, and thus keep themselves tension-free.

“*Even though the work was more satisfying, the thing is that we could go serve them when PLHIV were not able to come and take their medicines. If you think it's a problem, it is a problem. If you don't think of it as a problem, it is not.”* 32, Nurse, AP-TS

“*When I got Covid, the other staff nurse, the pharmacist and whoever was there on duty in that time, they coordinated among themselves and with the doctor also helping us, they followed instructions on what to do, and like that we made sure that services were delivered to the patients.”* 46, Nurse, AP-TS

#### Relationships With Community

Most of the providers mentioned that their own difficulties during this crisis only heightened their sense of responsibility and commitment to the communities they work with. Being mindful of their responsibility for the communities they serve helped the providers remain motivated and overcome the many struggles during the pandemic.

In addition to providing emotional support and management of health problems for PLHIV, the providers described further extending their services to ensure the basic needs of the community were met. To that end, providers offered financial assistance to PLHIV thus ensuring they had money to travel and seek care when needed; teamed up with local NGOs and support networks to ensure that basic provisions were made available for communities. Working extra hours, providing support using the remuneration they received, and reaching out to communities to ensure services are delivered even when they were not designated to do the same are all testimonials to how deeply the providers cared for the communities.

Providers also mentioned that the support and understanding gesture from communities, also helped them do their duties better and with much more motivation.

“*Even if they leave for their villages, they stay connected to us via phones and we tell them when to visit the office, where to collect the medicines etc. We are doing all this using our own money. I am myself HIV positive and as I am going through this phase in life, I don't want others to suffer from this.” 42, NGO Counsellor, MH*

“*I think it's our duty to take care of the patient and we have to adapt ourselves to the situation and we have to provide the basic things, some of them didn't have food to eat and they didn't have money to travel... Most of them were becoming unemployed during that time., so we used to deposit some amount in their accounts, and then they used to come and visit.” 47, ART Doctor, KR*

“*I know that ART will be available to them. But before consuming ART one has to eat something so we mentioned their names as per priority in the grocery distribution list. I have arranged for this work beforehand only.” 42, NGO Staff, MH*

“*I give them moral boosting to remain strong psychologically and rise up to the situations. I get calls till 10 pm at times. I have been counseling for 20 years and I dedicate my life for this purpose.” 49, ICTC Counsellor, AP-TS*

#### Collaborations With a Network of Providers

As the name suggests, the third sub-theme describes how the providers formed strong networks with the local providers to enhance their crisis response and continue service provision.

Even though the institutions that the providers in our study represent are self-sufficient in providing services for PLHIV, the challenges posed by the pandemic made it extremely difficult to continue care delivery. Study respondents mentioned that interdisciplinary collaboration is essential to provide quality care and efficient services. This led them to make quick adaptations to their service delivery methods as information regarding COVID-19 management evolved.

“*In this situation of COVID, we try to deliver medicines at the doorstep of patients who do not have enough money or cannot travel to the center. We take help from NGOs, CBOs, TI, and VIHAN. This is a chain, where all have to work in coordination then only, we can see progress.” 42, Counselor, MH*

It was reported that civil society organizations partnered with National AIDS control societies and programs and public health facilities joined forces with NGOs; to identify the beneficiaries, distribute medicines and provisions with the support of outreach workers (ORWs) as well as create awareness and communicate the availability of services.

“*During the lockdown period, the patients could not come here at all. So, what we did was we made a micro plan with the Share India team, Naari Shakti, these NGOs. So we took the outreach workers and with a list of the patients and their medicines details, they went on a truck and did home delivery of medicines.” 35, ART Doctor, AP-TS*

Providers also mentioned that because of these linkages, they were able to overcome the challenges due to the lack of transport facilities. Working side by side with local hospitals and health centers enabled providers to use institutional vehicles for conducting outreach. It was also discussed that informing the local government authorities about the nature of their job helped them procure permissions and travel passes which helped mitigate the troubles caused by interactions with police.

“*We used the TANSACS hospital vehicle to supply the drugs to the center and ensured that it reached the PLHIV patients.” 37, Program Staff, TN*

‘*'Yes police stopped us at times but that was not a big problem as now we had our identity cards and official permission from government authorities as healthcare providers.”* 49, Counsellor, AP-TS

Forming alliances with care providers at multiple levels ensured that there was no break in the provision of services. In addition to verifying whether patients were receiving care from tertiary hospitals and other primary health facilities locally, these coalitions also made it possible to deliver care to beneficiaries from other states as well.

“*We made a list of patients who were at their native and also the list of contact details of centers at various locations and informed the centers. We informed the patients to collect medicines from wherever they are and asked them to inform us in case the centers didn't provide them medicines so that we can recommend them directly.” 39, ART Nurse, KR*

## Discussion

This study explored healthcare provider experiences while providing HIV care during the first wave of COVID-19 in India. Our findings highlight the extensive obstacles that providers faced, despite which, they remained resilient and continued providing quality care to PLHIV by adopting novel and innovative strategies. The concept of assessing health systems from a resilience perspective is recent, where capacities of health actors and institutions are viewed from their ability to prepare, absorb and recover from unexpected and dynamic shocks and situations in the most equitable manner (17). Prevalent throughout the narrative of our study participants was a sense of responsibility and the need for coordinated efforts to ensure care is provided for the communities they serve. Our HIV care providers lent to situational resilience (18) by providing telephonic consultations, door-to-door delivery of medications, and collaborating with local NGOs and community organizations.

The respondents in our study faced several challenges- ranging from systemic, community-level, and personal, which resulted in high levels of stress and anxiety. These stresses need to be viewed in conjunction with the disease burden of COVID-19 in India and the capacity of the health system, to better understand the results. Although a few respondents mentioned the practice of meditation, seeking family support, and initiation of team-building activities in the workplace, our findings highlight the burden of mental health issues the providers in our study were facing and that none of them recognized the need to seek out help. To prevent burnout, an immense need exists for provider-supportive work environments and affordable formal mental healthcare services since previous studies reflect that such environments help exhibit greater resilience, including emotional regulation, self-efficacy, and adaptive coping strategies in providers. Being resilient psychologically and responding to situations appropriately is important for healthcare employees, especially when facing crisis situations like the COVID-19 pandemic. It also enables the providers to overcome the multitude of challenges as well as recover from the pandemic more easily (19).

Aside from workplace interventions, we believe that financial and personal security would also play a key role in lowering stress for providers and helping them provide quality care. Our study respondents reported that most of them were not given remuneration for their services for months, most did not receive transport allowance, and some did not even have adequate PPE to protect themselves from COVID-19. Socio-economic deprivation, such as this, can have a long-term impact on mental health and delay care-seeking among providers (20).

Health system financing has been a long-term cause for concern in India (21) and though the one-time support to communities and providers in the form of monetary relief and insurance support by the government is extremely important, it does not lend to structural impact on the health system efficiency and support (22). Long-term resilience needs to prioritize the economic security and social protection that are mindful of provider challenges.

Our study highlights areas of action that can be undertaken to provide and enhance systemic resilience for future resilience preparedness. While the providers could use their personal relationships and networks to build and provide to the continuum of care for PLHIV, a government led-multisectoral approach that includes all levels of stakeholders, including local authorities and communities (23) based on clear and coordinated SOPs is required for enhanced long-term management for emergencies. Further, bolstering public health capacity through an increase in the skilled and protected healthcare workforce and continual training support will provide strengthened linkages in the health system.

Our study comes at a time when it is imperative to realize the toll that COVID-19 is taking on our healthcare system and its repercussions among the provider community. The findings from our study are not unique, however, they highlight the long-standing problems of our health system that were exposed and severely compromised during COVID-19. What is unique, however, is the resiliency that was provided due to the initiatives taken by frontline workers to care for the most vulnerable. The strength of the findings lies in the adaptability and agile response that the providers were organically able to provide, which makes systemizing them a potential pandemic response. Despite our best efforts, our study has some limitations. Since the findings are self-reported, respondents may be influenced by the poor recall and social desirability bias. Further, the data we have is specific to five Indian states and is limited to experiences of providers catering to PLHIV during the first wave of COVID-19. Therefore, the findings may differ with geographies and the intensity of subsequent waves of the pandemic and may not capture systemic issues across the entire healthcare system. In addition, because of the ongoing restrictions like physical distancing and other infrastructural challenges, we conducted the interviews through the telephone; which may have resulted in the loss of a more nuanced interpretation of the findings from the body language of the participants, etc.

Even though the providers strived to remain resilient and continued providing quality care for PLHIV, the sudden change in the work culture and intensive work have drained health care providers emotionally and physically. It is important to conduct rigorous studies to understand the impact of the pandemic on the psychosocial wellbeing of healthcare providers, which will throw light on the role of regulators like resilience etc. Government should also take a holistic approach to safeguard the wellbeing of providers and invest in efficient systems and disaster preparedness to manage any future crises.

## Data Availability Statement

The raw data supporting the conclusions of this article will be made available by the authors, without undue reservation.

## Ethics Statement

The studies involving human participants were reviewed and approved by Catalysts Group Institutional Review Board. Verbal consent was sought from all participants in the study in accordance with the national legislation and institutional requirements.

## Author Contributions

NP, SB, and PS wrote the first draft of the manuscript. AC critiqued the final version. All authors contributed to the study conception and design, material preparation, data collection and analysis, commented on the latter versions of the manuscript, and read and approved it.

## Conflict of Interest

NP, AC, SS, and PS were employed by Swasti.

## Publisher's Note

All claims expressed in this article are solely those of the authors and do not necessarily represent those of their affiliated organizations, or those of the publisher, the editors and the reviewers. Any product that may be evaluated in this article, or claim that may be made by its manufacturer, is not guaranteed or endorsed by the publisher.

## Footnotes

^1^Swasti is a not-for-profit organization headquartered in Bangalore established in 2002, focusing on achieving health outcomes, especially for socially excluded and marginalized communities.
